# Graphene-Skinned Materials: Direct Integration Strategies, Structural Insights, and Multifunctional Applications

**DOI:** 10.3390/nano15211679

**Published:** 2025-11-05

**Authors:** Yulin Han, Xinya Lu, Ningning Su, Yingjie Zhao, Qingyan Pan

**Affiliations:** 1 School of Semiconductor and Physics, North University of China, Taiyuan 030051, China; 2 College of Polymer Science and Engineering, Qingdao University of Science and Technology, Qingdao 266000, China; 2024022058@mails.qust.edu.cn (X.L.); yz@qust.edu.cn (Y.Z.)

**Keywords:** graphene, graphene-skinned materials, chemical vapor deposition

## Abstract

Graphene, owing to its unique atomic structure, exhibits a set of outstanding physical and chemical properties, including ultrahigh carrier mobility, excellent thermal conductivity, superior mechanical strength, and high optical transparency. However, the atomic-thickness nature of graphene limits its ability to form self-supporting structures, making substrate integration a prerequisite for practical applications. Graphene-skinned materials, constructed by in situ deposition of continuous graphene films on conventional substrates, have recently emerged as a promising solution. This strategy effectively integrates graphene with conventional engineering materials, harnessing its superior properties while avoiding the structural defects and contamination typical of transfer processes. Consequently, graphene-skinned materials have rapidly become a rapidly developing area of research in materials science. This review systematically summarizes recent advances in graphene-skinned materials. Particular attention is given to coating methods and chemical vapor deposition (CVD) routes, followed by a discussion of commonly employed characterization tools for evaluating graphene quality and interface integrity. Applications in electromagnetic shielding, thermal management, sensors, and multifunctional composites are critically examined. Finally, future perspectives are needed regarding the key challenges and opportunities for engineering and industrial-scale deployment of graphene-skinned materials.

## 1. Introduction

Since the successful isolation of monolayer graphene via mechanical exfoliation by Geim and Novoselov in 2004, graphene has rapidly risen to the forefront of materials science [1]. Owing to its two-dimensional honeycomb lattice, graphene displays a range of exceptional properties: carrier mobility as high as 2 × 10^5^ cm^2^ V^−1^·s^−1^; a zero-bandgap Dirac-cone electronic structure with ultrahigh conductivity and room-temperature quantum Hall effect [2,3]; theoretical tensile strength of ~130 GPa and Young’s modulus of ~1 TPa [4]; thermal conductivity approaching 5000 W·m^−1^ K^−1^ [5,6]; and optical transmittance of 97.7% in the visible region [7]. These attributes endow graphene with enormous potential in flexible electronics, advanced thermal management, and optoelectronic devices.

Nevertheless, the translation of graphene from laboratory research to practical applications remains challenging. Scalable synthesis of large-area, defect-free graphene with controlled thickness and uniformity has yet to be achieved. The atomic-thin morphology lacks mechanical robustness, making it difficult to integrate with device substrates or assemble into three-dimensional structures. State-of-the-art CVD techniques typically require transfer from catalytic metal substrates to functional supports, which is a process that frequently induces cracks, wrinkles, contamination, and other structural imperfections [8]. As a result, the intrinsic properties of graphene are often compromised at the device level, impeding reproducibility and industrial viability.

Graphene-skinned materials can be viewed as a unique subclass within the family of graphene-based hybrid architectures. Unlike composites or heterostructures that typically rely on dispersed graphene fillers or laminated assemblies, graphene-skinned materials feature an atomically continuous coating directly bonded to the substrate. This configuration ensures efficient interfacial coupling and defect-free integration, thereby maximizing the utilization of graphene’s intrinsic conductivity, thermal transport, and mechanical strength within a macroscopic framework.

**Figure 1:**
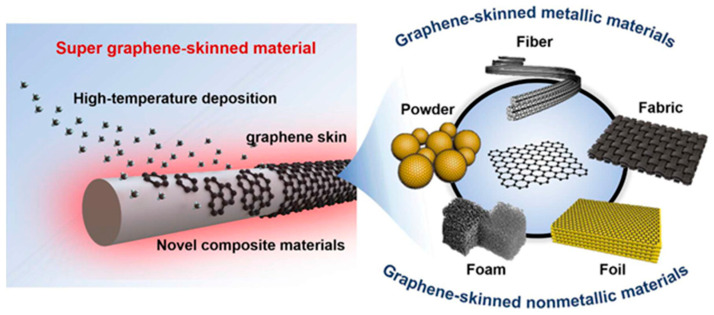
Schematic illustration of the graphene “skin” formed on various substrates. Reproduced with permission from Ref. [9]. Copyright 2024, American Chemical Society.

To overcome the limitations of transfer and structural discontinuity, an emerging strategy is the development of graphene-skinned materials—continuous, high-quality graphene films grown directly on non-metallic or conventional substrates (e.g., glass fibers, ceramics, polymers) (Figure 1) [9]. This in situ “skin” growth strategy eliminates the need for transfer steps, maximally retaining the intrinsic integrity of graphene while ensuring robust adhesion to the substrate. Compared with the physical coating of graphene flakes, graphene skins provide continuous lattice structures with superior electrical and mechanical integrity, significantly enhancing the mechanical strength, conductivity, thermal transport, and chemical resistance of the host substrate. Furthermore, the macroscopic morphology and mechanical support of the base material improve the processability, design flexibility, and service stability of graphene, combining the exceptional properties of two-dimensional graphene with the structural robustness of conventional materials.

Graphene-skinned materials have been successfully fabricated on various substrates, including fibers, powders, foils, and foams [9]. Such materials have demonstrated promising performance in electromagnetic shielding, heat dissipation, and electrochemical energy storage, highlighting their potential for flexible electronics, multifunctional composites, and energy technologies. Despite this progress, the field remains in its infancy, with several scientific and engineering challenges yet to be addressed. A systematic understanding of graphene-skinned materials—bridging direct growth mechanisms with functional performance—remains missing. Moreover, understanding the initial stages of graphene nucleation and growth is critical for achieving high-quality films suitable for high-performance devices. Modeling and theoretical studies have provided valuable insights into the adsorption behavior of carbon-containing molecules on catalytic surfaces, which governs the nucleation density, domain orientation, and growth kinetics in CVD processes [10,11]. Such investigations are particularly relevant for Cu and Si substrates, revealing how precursor adsorption energetics and surface interactions influence graphene layer formation. Additionally, low decomposition-barrier carbon precursors, such as toluene, have been demonstrated to facilitate efficient CVD growth by increasing the availability of active carbon species, enabling better control over domain size, defect density, and layer uniformity. These theoretical and experimental insights complement empirical CVD optimization, providing a more complete understanding of the synthesis process and guiding the fabrication of graphene-skinned materials with superior structural and functional properties.

In this review, we provide a comprehensive overview of the preparation, characterization, and applications of graphene-skinned materials. We highlight advances in CVD and other direct-growth strategies on non-metal substrates, elucidate the structure–property relationships, and evaluate prospects for energy conversion and storage, flexible devices, sensors, and structural–functional composites. Finally, we outline the opportunities and critical challenges that must be overcome for the large-scale engineering and industrial implementation of graphene-skinned materials.

Importantly, this review distinguishes itself from the existing literature by providing a focused and systematic analysis of graphene-skinned materials as a distinct class within graphene-based composites. While numerous reviews have addressed graphene composites, heterostructures, or filler-based architectures, few have comprehensively examined the unique advantages of continuous, substrate-integrated graphene films. Here, we critically summarize both the synthesis strategies and the structure–property relationships that govern performance, highlight recent advances in direct-growth techniques on non-metallic substrates, and provide a detailed discussion of applications where graphene-skinned architectures outperform conventional composites. By bridging mechanistic understanding, materials design, and functional implementation, this review aims to offer a valuable roadmap for researchers and engineers seeking to develop high-performance graphene-skinned materials and translate them into scalable, application-ready technologies.

For clarity and ease of reading, this review is organized as follows:

Section 2: Preparation Strategies of Graphene-Skinned Materials: Overview of CVD and alternative direct-growth methods for graphene-skinned materials.

Section 3: Characterization of Graphene-Skinned Materials: Structural, morphological, and functional characterization methods.

Section 4: Applications of Graphene-Skinned Materials: Key applications in thermal management, electromagnetic shielding, flexible electronics, optical devices, sensors, and electrothermal systems are reviewed, with comparisons to conventional graphene-based composites.

Section 5: Outlook: We discuss current bottlenecks, scalability, reliability, and industrial translation, highlighting directions for future research and development.

## 2. Preparation Strategies of Graphene-Skinned Materials

The fabrication of graphene-skinned materials has been explored through several complementary strategies, which can be broadly classified into solution-based coating, graphene transfer, and direct in situ CVD. Among these, CVD remains the most widely adopted approach due to its ability to deliver large-area, continuous graphene layers with controllable thickness and superior quality. Nevertheless, other solution-based and hybrid methods also play a critical role in tailoring graphene skins for specific substrates and applications.

### 2.1. Solution-Based Coating Methods

Solution-based coating represents one of the most straightforward strategies for fabricating graphene-skinned materials [12]. Graphene oxide (GO), reduced graphene oxide (rGO), or liquid-phase exfoliated graphene nanosheets are dispersed in solvents and deposited onto substrates via techniques such as dip coating, spin coating, spray coating, or vacuum filtration [13]. Typically, the solution-based coating approach for graphene is a material fabrication strategy following an “oxidation–reduction” route. The core process involves the chemical oxidation of graphite to obtain GO, ultrasonic exfoliation to form a stable GO dispersion, and subsequent chemical reduction or thermal treatment to produce rGO coatings. Multiple techniques can be applied within this strategy. Vacuum filtration enables the deposition of large-area films with controllable thickness and uniformity, while also allowing convenient transfer through the selective retention of nanomaterials, making it a versatile solution-processed method [14]. Spin-coating is suitable for creating uniform films on flat substrates [15]. Spray-coating allows rapid coverage over substrates with complex surface morphologies [16]. Dip-coating is widely used for the continuous processing of fibers or porous materials [17,18].

**Figure 2:**
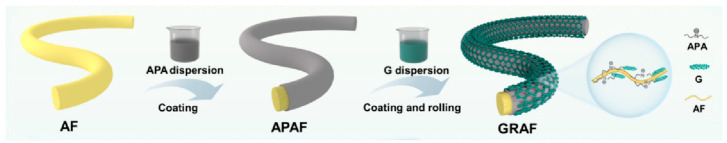
Schematic illustration of the fabrication process of graphene-coated aramid fibers (GRAF). Reproduced with permission from Ref. [18]. Copyright 2024, American Chemical Society.

Although the coating method has advantages such as facile operation, low cost, and scalability, the resulting rGO films generally exhibit structural defects and residual functional groups, leading to insufficient electrical conductivity. Furthermore, the poor stability of GO dispersions and the re-aggregation of nanosheets during reduction remain pressing challenges. To address these issues, researchers have significantly improved coating performance through interfacial modification and process optimization. For instance, Zhang et al. employed aramid polyanion (APA) as a multifunctional interfacial mediator and realized high-quality graphene encapsulation on aramid fibers (AF) via a two-step dip-coating strategy, thereby constructing structurally integrated graphene-coated aramid fibers (GRAF) (Figure 2) [18]. In this process, the APA solution, synthesized by deprotonation, was first coated onto AF surfaces. Owing to its high surface energy (55.2 mJ/m^2^), APA could micro-etch the smooth AF surface to create groove-like features that enhance roughness, thus providing more anchoring sites for graphene. Molecular dynamics simulations revealed that the binding energy between APA and graphene (1.3 J/m^2^) was significantly higher than that between AF and graphene (0.2 J/m^2^), confirming APA as an effective “molecular bridge” to reinforce interfacial interactions. Subsequently, the APA-pretreated fibers were immersed in an optimized graphene dispersion (2 mg/g), where π–π stacking, mechanical interlocking, and surface energy matching collectively drove the oriented self-assembly and compact encapsulation of graphene. After heat treatment, washing, and rolling, dense and firmly bonded GRAF was obtained. This strategy not only enhanced graphene adsorption and orientation but also effectively mitigated solvent residue and nanosheet aggregation. As a result, GRAF exhibited outstanding electrical conductivity (1062.04 ± 116.78 S/m) and superior mechanical properties (tensile strength: 4.66 ± 0.16 GPa; modulus: 106.33 ± 8.21 GPa).

While solution-based methods are effective for laboratory-scale demonstrations, their ability to produce high-quality, defect-free, large-area graphene films is limited compared with CVD. Future studies could focus on combining solution processing with mild thermal or chemical treatments to balance scalability and film quality.

### 2.2. Direct Transfer Method

**Figure 3:**
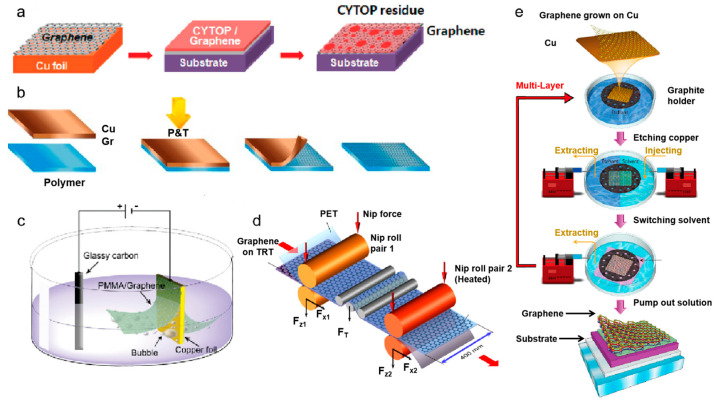
(**a**) Schematic diagram of the wet transfer process. Reproduced with permission from Ref. [23]. Copyright 2012, American Chemical Society. (**b**) Dry transfer process. Reproduced with permission from Ref. [24]. Copyright 2014, Elsevier. (**c**) Bubble transfer process. Reproduced with permission from Ref. [25]. Copyright 2011, American Chemical Society. (**d**) Roll-to-roll transfer process. Reproduced with permission from Ref. [26]. Copyright 2017, IOP Science. (**e**) Support-free transfer process. Reproduced with permission from Ref. [27]. Copyright 2014, American Chemical Society.

The transfer of pre-grown graphene films, particularly those synthesized via CVD on catalytic metal foils, has also been widely used to construct graphene-skinned materials [19,20]. The mostly used graphene transfer methods include wet transfer, bubble-mediated transfer, dry transfer, roll-to-roll transfer, and support-free transfer [21]. Wet transfer involves chemically etching the metal substrate and transferring the graphene film to the target substrate, which is widely used in laboratory research but prone to polymer residue and etchant contamination that degrade graphene performance (Figure 3a) [22,23]. For example, Leong et al. [22] developed a paraffin-assisted transfer method that yielded high-quality, wrinkle-free, and clean large-area graphene films. In this approach, paraffin served as a support layer with a high thermal expansion coefficient and low chemical activity. Heating in 40 °C water allowed paraffin expansion to stretch graphene and eliminate wrinkles, followed by organic solvent cleaning to achieve a clean surface and uniform electrical properties. Dry transfer employs thermal-release tapes or flexible polymer films to mechanically peel graphene without liquid contact, though mechanical stress may induce cracks (Figure 3b) [24]. Bubble transfer, which relies on gas bubbles generated via chemical reactions to delaminate graphene, enables substrate reuse and rapid transfer but is limited to conductive substrates and requires precise control of process parameters (Figure 3c) [25]. Roll-to-roll transfer enables large-area, continuous transfer and lamination via a continuous rolling process, demonstrating industrial potential but still facing challenges in maintaining uniform coverage and minimizing damage (Figure 3d) [26]. Support-free transfer, relying on electrostatic adsorption or hot pressing, avoids polymer contamination but has low success rates and higher risks (Figure 3e) [27].

Despite extensive progress, graphene transfer technologies still face key challenges: contamination and structural damage cannot be completely avoided. In particular, polymer residues and metal ion doping significantly degrade electrical properties. Current post-transfer cleaning treatments are costly, time-consuming, and environmentally taxing, yet defects may persist even after cleaning, restricting commercialization [14,20,28,29,30]. Furthermore, complex multi-step processes and high costs remain major barriers to industrial adoption. Given these limitations, direct-growth approaches that eliminate the transfer step are increasingly emphasized. In particular, direct graphene growth on functional substrates (e.g., semiconductors or metal oxides) offers significant advantages by avoiding transfer-induced defects and contamination, and represents a promising research direction for advancing graphene-based device applications [31,32,33].

Transfer methods are effective for research and small-scale applications, but the persistent challenges of contamination, defect formation, and low reproducibility suggest that direct-growth approaches may be more suitable for large-area, high-performance devices. Comparative studies quantifying the impact of transfer-induced defects on device performance could strengthen the understanding of practical limitations.

### 2.3. Direct In Situ Chemical Vapor Deposition

**Figure 4:**
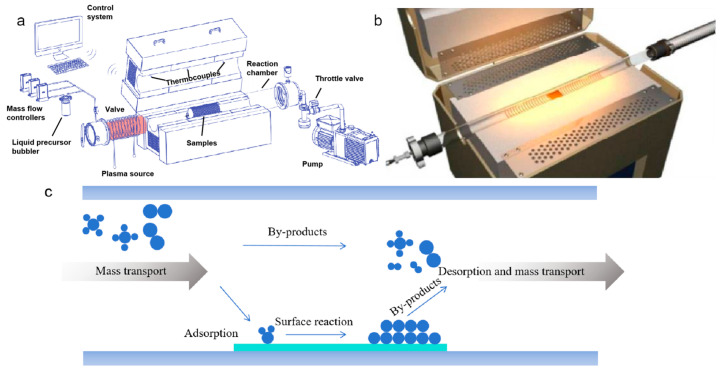
(**a**,**b**) Schematic and photograph of the CVD setup. Reproduced with permission from Ref. [36]. Copyright 2021, Springer Nature. Reproduced with permission from Ref. [37]. Copyright 2014, American Chemical Society. (**c**) Illustration of the elementary steps in a typical CVD process.

In situ CVD growth directly on target substrates has become the most promising and intensively studied method for preparing graphene-skinned materials, as it eliminates the need for post-growth transfer and ensures intimate interfacial bonding (Figure 4a,b). CVD is a method in which gaseous carbon precursors decompose under high-temperature or external field conditions, followed by surface deposition of carbon atoms to form graphene films (Figure 4c) [34,35].

On metal substrates, the growth mechanism largely depends on the dissolution, diffusion, and nucleation behavior of carbon species catalyzed by the metal. For metals with high carbon solubility (e.g., Ni [38,39]), carbon atoms dissolve into the bulk metal at elevated temperatures and precipitate to form graphene layers during cooling [40]. In contrast, for low-solubility metals (e.g., Cu), carbon atoms adsorb, diffuse, and nucleate mainly on the surface. Once graphene covers the surface, further reactions are suppressed, leading to self-limited growth, which favors the formation of uniform monolayer films. The metal substrate thus not only lowers the decomposition barrier of carbon precursors but also regulates graphene domain size, orientation, and film uniformity through its lattice structure and surface morphology, thereby directly influencing electrical and mechanical properties. In recent years, CVD growth of graphene on metal substrates has become increasingly mature in terms of crystal quality and industrial-scale production [41,42,43,44].

Recent progress has also been made in the direct growth or chemical anchoring of graphene layers on metallic and composite substrates. For example, CVD-grown graphene on Cu, Ni, or Fe-based alloys has demonstrated improved charge transport and interfacial electron coupling, enabling enhanced electrochemical performance in corrosion protection, electrocatalysis, and energy storage devices. Similarly, chemical anchoring of graphene onto metal composites or conductive ceramics has been shown to reduce interfacial resistance and suppress electron scattering, thereby improving overall conductivity and mechanical robustness. These studies highlight the crucial role of strong graphene–substrate interactions in modulating charge transfer dynamics and interfacial stability. However, metal-based systems remain limited by potential catalyst diffusion, interfacial contamination, and transfer-related defects. In this context, direct in situ CVD growth of graphene “skins” on non-metallic substrates provides an effective alternative, ensuring clean interfaces and defect-minimized integration.

**Figure 5:**
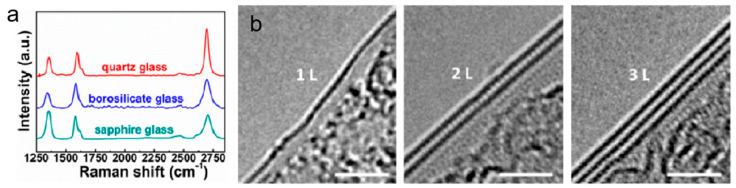
(**a**) Raman spectra of graphene directly grown on various glass substrates. (**b**) HRTEM images of monolayer, bilayer, and trilayer graphene. Scale bar: 2 nm. Reproduced with permission from Ref. [50]. Copyright 2015, American Chemical Society.

Unlike the conventional transfer processes on metal substrates, graphene-skinned materials can be fabricated by directly growing graphene “skins” in situ on non-metallic or insulating substrates via CVD strategies, achieving intimate integration between graphene and the underlying substrate. This approach not only eliminates wrinkles, cracks, and contamination that may arise during transfer but also preserves the intrinsic properties of graphene, such as high electrical conductivity, excellent mechanical strength, and optical transparency. Nevertheless, direct CVD growth of graphene on non-metallic or insulating substrates faces numerous challenges. The decomposition of carbon precursors strongly relies on high temperatures (typically >1000 °C), while the high migration barrier of carbon atoms often leads to random multi-point nucleation, resulting in poor uniformity in layer number and thickness, as well as limited growth rates. To overcome these issues, high-temperature-resistant substrates such as quartz glass [45], sapphire [46], alumina [47], silicon carbide [48], and hexagonal boron nitride [49] can be employed, combined with optimized catalyst-free atmospheric pressure CVD processes to achieve large-area uniform graphene films. By precisely tuning growth temperature, gas composition, and carbon precursor flow rate, continuous lattice structures can be preserved while enabling accurate modulation of film properties. For example, Sun et al. developed a catalyst-free atmospheric pressure CVD method that successfully realized large-area, uniform graphene films on solid glass substrates with high thermal stability (e.g., quartz and sapphire glass) (Figure 5a), thereby producing graphene-glass composites with outstanding optoelectronic performance [50]. Through controlling methane flow rate, growth time, and temperature, precise regulation of graphene layer number, optical transparency, and sheet resistance can be achieved (Figure 5b). For instance, at a wavelength of 550 nm, the graphene-glass exhibited a transmittance of up to 97.5% with a corresponding sheet resistance of 6.1 kΩ sq^−1^; at a transmittance of 80%, the sheet resistance decreased to as low as 2 kΩ sq^−1^, surpassing most reduced graphene oxide and plasma-enhanced CVD-derived samples.

In addition, selecting carbon precursors with lower decomposition barriers (e.g., ethanol [51], acetylene [52], and propane [53]) can increase the concentration of active carbon species, thereby significantly accelerating the graphene growth rate and facilitating the control of grain size and defect density. However, on non-catalytic substrates, issues such as multiple nucleation events, slow domain coalescence, and insufficient film uniformity remain to be addressed, indicating that the rapid and high-quality growth of graphene still holds broad potential for optimization. Chen et al. [54] developed a confined-flow CVD approach, in which a restricted gas channel of 2–4 μm was constructed above the glass substrate with barrier structures. This design markedly enhanced the collision probability of carbon precursors with the glass surface and increased the local concentration of reactive species, thereby enabling the rapid and uniform growth of high-quality graphene films directly on conventional glass substrates. Using this method, graphene preparation could be completed within 75 min, with both growth rate and film quality (e.g., a significantly reduced D/G peak intensity ratio) surpassing those achieved by conventional flow CVD techniques.

**Figure 6:**
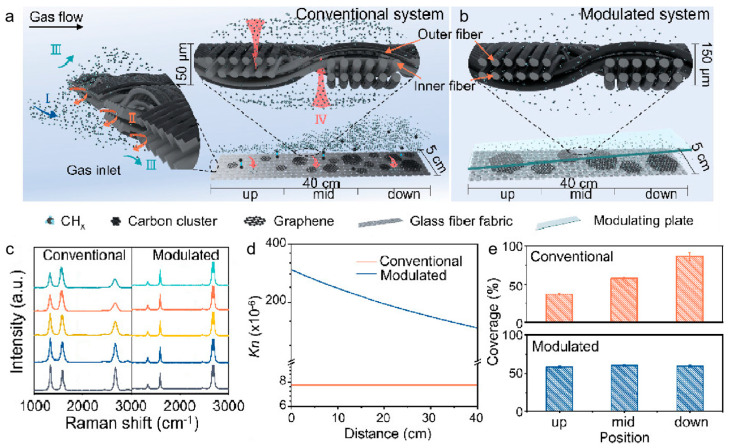
(**a**,**b**) Growth of graphene on GGFF using a conventional CVD system (**a**) and the fluid-dynamics-rectified CVD system (**b**). (**c**) Raman spectra of graphene on GGFF along the gas flow direction (from bottom to top). (**d**) Theoretical calculation of the Knudsen number (Kn) of gas molecules on GGFF along the flow direction. (**e**) Graphene coverage under identical growth conditions. Reproduced with permission from Ref. [55]. Copyright 2024, American Chemical Society.

Yang et al. [55] developed a fluid-dynamics-rectified CVD system, in which angled modulating plates were positioned above a three-dimensional glass fiber fabric to create a confined space with a gradient along the gas flow direction (Figure 6a,b). On one hand, this configuration transformed gas transport from convection-dominated to diffusion-dominated flow, significantly enhancing the spatial uniformity of active carbon species both inside and outside the fabric. On the other hand, the progressively decreasing Knudsen number (Kn) along the gas flow direction effectively compensated for the concentration gradient of reactive carbon species caused by continuous precursor decomposition, leading to a more homogeneous deposition rate throughout the three-dimensional architecture (Figure 6e). Experimental results (Figure 6c) confirmed that this strategy reduced graphene defect density, improved control over the layer number, suppressed carbon cluster formation, and promoted the ordered nucleation of carbon atoms on the substrate surface. Furthermore, this dynamic compensation mechanism exhibited excellent generality—it can be tuned by adjusting the modulating plate angles to accommodate different carbon precursors with distinct decomposition behaviors, thus providing both a theoretical foundation and a practical pathway for scalable, transfer-free growth of high-quality graphene-skinned glass fiber fabrics (GGFF).

Although CVD offers significant advantages for the fabrication of graphene-skinned materials, several challenges remain. On non-catalytic substrates, random multiple nucleation events frequently occur, resulting in non-uniform layer distribution; the high temperatures required limit the applicability to certain thermally sensitive substrates; and restricted carbon atom migration and domain coalescence rates hinder the rapid formation of large-area, high-quality films. To address these issues, current research has focused on optimizing substrate surface properties, introducing localized catalysts or external field assistance, and improving carbon precursor design. Overall, the CVD method provides an effective route for the high-quality preparation of graphene-skinned materials [43]. Its continuous lattice structure and strong interfacial bonding endow it with broad application potential in flexible electronics, transparent conductive films, high-performance composites, and functional surfaces.

## 3. Characterization of Graphene-Skinned Materials

The characterization of graphene-skinned materials primarily focuses on evaluating the growth quality of the graphene overlayer, which directly reflects its growth behavior and interfacial bonding on different substrates. Spectroscopic and microscopic techniques are typically employed to systematically probe the morphology, structural features, defect density, and layer number of the graphene coating. Among them, Raman spectroscopy, scanning electron microscopy (SEM), transmission electron microscopy (TEM), and atomic force microscopy (AFM) serve as the most widely used tools. Together, these techniques establish a multi-scale and multi-dimensional analytical framework, providing essential insights into the structure–property relationships of graphene–substrate hybrid systems.

**Figure 7:**
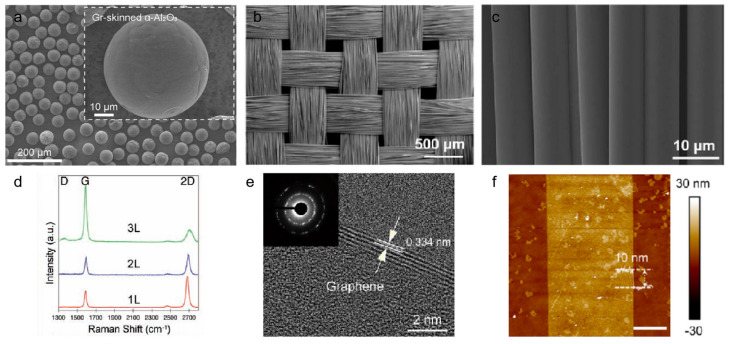
(**a**–**c**) SEM images of graphene-skinned Al_2_O_3_ powders, GGFF, and GGF. Reproduced with permission from Ref. [62]. Copyright 2025, Wiley-VCH GmbH. Reproduced with permission from Ref. [9]. Copyright 2024, American Chemical Society. (**d**) Raman spectra of graphene with different layer numbers. Reproduced with permission from Ref. [43]. Copyright 2009, American Association for the Advancement of Science. (**e**) TEM images and the corresponding SAED patterns. Reproduced with permission from Ref. [47]. Copyright 2025, Tsinghua University Press. (**f**) AFM topography of graphene layers. Reproduced with permission from Ref. [63]. Copyright 2022, American Chemical Society.

Scanning electron microscopy (SEM) is a key technique for probing the surface morphology, microstructure, and topography of graphene layers. With its high-resolution imaging capability, SEM provides direct evidence of graphene coverage uniformity, layer continuity, defect distribution, and interfacial interactions with the substrate [56]. It is also useful for evaluating whether substrates undergo morphological changes during high-temperature CVD growth. As shown in Figure 7a–c, graphene uniformly and conformally coats both Al_2_O_3_ powders and glass fiber fabrics without significant morphological alteration, demonstrating the structural stability of the fabrication process. SEM has thus been widely applied in growth mechanism studies, contamination assessment, and preliminary layer-number determination of graphene.

Raman spectroscopy serves as the most effective tool for correlating graphene quality with growth conditions, offering quantitative insights into defect density and layer thickness. Typical graphene Raman spectra feature three peaks: the D band (~1350 cm^−1^) associated with defect-induced phonon scattering [57], the G band (~1580 cm^−1^) corresponding to the in-plane vibration of sp^2^ carbon atoms [58], and the 2D band (~2700 cm^−1^) arising from a double-resonance process. The intensity ratio ID/IG is widely adopted to evaluate defect density, while the shape, position, and I2D/IG ratio of the 2D band serve as reliable fingerprints for distinguishing monolayer, few-layer graphene, and graphite (Figure 7d) [59].

Transmission electron microscopy (TEM) and selected-area electron diffraction (SAED) provide complementary insights into atomic structure and crystallinity. TEM enables atomic-resolution imaging of graphene’s lattice, bonding configuration, and defect structures. For instance, Chee-Tat Toh et al. visualized continuous but distorted carbon ring networks in monolayer amorphous carbon, highlighting its distinct topology compared with crystalline graphene [60]. SAED patterns further discriminate crystalline from amorphous states. Crystalline graphene exhibits sharp diffraction spots or rings, while amorphous carbon shows diffuse halos. Figure 7e illustrates TEM and SAED results of graphene-skinned materials, revealing few-layer stacking with bright diffraction spots indicative of high crystallinity. Together, TEM and SAED bridge atomic-scale imaging with long-range structural order, providing decisive evidence of graphene quality.

Atomic force microscopy (AFM) is a standard approach for thickness determination, offering nanoscale resolution of surface topography [61]. However, AFM-measured thicknesses often exceed the theoretical 0.34 nm per graphene layer, mainly due to surface adsorbates, tip–sample interactions, and environmental factors. For graphene-skinned materials on complex substrates such as fabrics or powders, direct AFM measurement is particularly challenging. A common strategy involves substrate removal: Yang et al. [32] etched glass fibers with HF solution to release free-standing graphene ribbons, which were subsequently transferred onto Si substrates for AFM profiling. As shown in Figure 7f, AFM images revealed surface roughness and thickness of graphene layers with high clarity, providing reliable information on layer number and coverage.

SEM, Raman, TEM/SAED, and AFM constitute a comprehensive multi-scale toolkit for evaluating graphene-skinned materials. These techniques together enable correlation between growth conditions, interfacial binding, and ultimate material properties, thereby offering critical insights into structure–property relationships in graphene-based composite systems. While existing characterization techniques provide comprehensive structural insights, integrating in situ monitoring (e.g., real-time Raman, environmental TEM) could further elucidate growth dynamics and defect formation. A critical evaluation comparing different characterization methods’ sensitivity, resolution, and practical applicability would add value to readers aiming to optimize fabrication processes.

## 4. Applications of Graphene-Skinned Materials

Graphene-skinned materials, featuring a unique “graphene coating” structure, effectively impart the high electrical conductivity, excellent thermal conductivity, and outstanding mechanical properties of graphene to conventional substrates. This design endows the materials with superior functional performance and structural stability while avoiding common defects and interfacial delamination associated with traditional graphene transfer processes. Benefiting from this structural strategy, graphene-skinned materials exhibit advantages such as light weight, high strength, and designable flexibility, along with excellent weather resistance, process compatibility, and scalability for large-area fabrication. These characteristics make them highly promising for applications in aerospace anti-icing, wind turbine blade protection, industrial high-efficiency heating, electromagnetic interference (EMI) shielding, and energy storage devices, representing a key direction in current graphene application research.

### 4.1. Thermal Management

The graphene films on the surface of graphene-skinned materials possess intrinsically high thermal conductivity, excellent mechanical properties, and outstanding thermal stability, making them ideal candidates for constructing efficient thermal management systems [64]. Compared with standalone graphene films, graphene-skinned materials directly grow graphene on the substrate surface, providing mechanical support for the graphene and establishing a three-dimensional continuous thermal conduction network. This architecture significantly reduces phonon scattering at interfaces, allowing graphene’s exceptional in-plane thermal conductivity to be fully realized.

**Figure 8:**
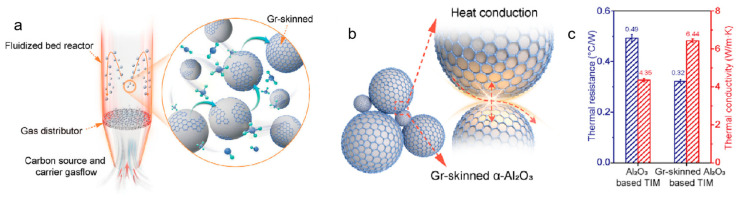
(**a**) Schematic diagram of the preparation process of graphene-skinned Al_2_O_3_ powders. (**b**) Schematic illustration of interfacial heat conduction. (**c**) Comparison of thermal conductivity and thermal resistance among different TIMs. Reproduced with permission from Ref. [62]. Copyright 2025, Wiley-VCH GmbH.

Wu et al. developed a fluidized-bed CVD synergistic process, as illustrated in Figure 8a, achieving controlled growth of continuous and highly crystalline graphene layers on α-Al_2_O_3_ powders with particle sizes of 5, 40, and 70 μm [62]. The resulting graphene-skinned powders exhibited over 99.5% surface coverage, forming a through-going “Phonon Expressway.” In solid nonmetallic materials, heat transfer mainly relies on phonons—the quantized units of lattice vibrations. Therefore, the concept of a “Phonon Expressway” vividly describes the efficient and rapid transport channels for thermal energy within a medium. Specifically, in the graphene/Al_2_O_3_ composite powder system, continuous graphene coating layers are constructed on the surfaces of alumina particles, forming an interconnected three-dimensional thermal conduction network. This structure provides a low-scattering and high-flux pathway for phonon transport, significantly enhancing phonon conduction efficiency within the system. As a result, macroscopic heat diffusion and thermal management are greatly improved. The thermal transport mechanism is depicted in Figure 8b. Heat conduction is dominated by the graphene layers and interlayer interfaces, with thermal flux across particle contact interfaces exceeding that of the Al_2_O_3_ substrate by more than an order of magnitude. The thermal interface materials (TIMs) based on graphene/Al_2_O_3_ were compared with pristine Al_2_O_3_ TIMs, as shown in Figure 8c. The graphene/Al_2_O_3_ TIMs exhibited a thermal conductivity of 6.44 W·m^−1^·K^−1^, representing a ~48% increase over the unmodified system (4.35 W·m^−1^·K^−1^), while thermal resistance decreased by approximately 53%. Moreover, after 500 thermal cycles between 40 and 100 °C, the thermal conductivity decay was less than 3%. This study not only demonstrates the significant enhancement of graphene-skinned materials in thermal management applications but also highlights the feasibility of the FB-CVD process for scalable production.

### 4.2. Electromagnetic Shielding

The continuous graphene films on the surface of graphene-skinned materials, when integrated with a quartz fiber substrate, form a synergistic conductive–dielectric architecture that exhibits excellent electromagnetic shielding performance. The high-density free carriers within the graphene layers interact strongly with incident electromagnetic waves, enabling interface reflection and energy dissipation. Simultaneously, the dielectric properties of the quartz fibers and the periodic woven topology of the fabric induce multiple scattering and polarization losses across interfaces, significantly enhancing shielding effectiveness. Compared with conventional metal-based shielding materials, this system is lightweight, flexible, and environmentally robust, making it suitable for complex curved surface applications.

**Figure 9:**
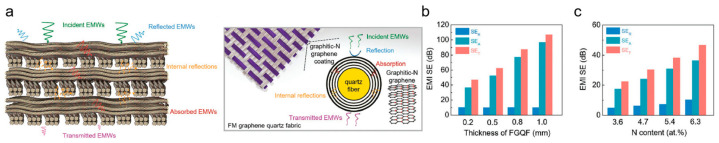
(**a**) Schematic diagram of EMI shielding mechanisms. (**b**,**c**) SET, SER, and SEA values of FGQF with different thicknesses and various N-substitutional levels. Reproduced with permission from Ref. [65]. Copyright 2022, Wiley-VCH GmbH.

Xie et al. employed a modulated doping CVD method to grow highly crystalline nitrogen-doped graphene in situ on quartz fabrics at 970–1050 °C, producing flexible, lightweight, and durable ferromagnetic graphene–quartz fabrics (FGQF) [65]. As illustrated in Figure 9a, the electromagnetic shielding mechanism of FGQF originates from its unique structural design: precise control of graphitic nitrogen doping (~6.3 at%) imparts high electrical conductivity (3906 S·cm^−1^) and ferromagnetism (saturation magnetization ≈0.14 emu·g^−1^ at 300 K). Coupled with the multiple interface topology of satin weave fabrics, this structure enables a synergistic shielding effect through repeated reflection and absorption of electromagnetic waves. As shown in Figure 9b, FGQF with a thickness of only 1 mm achieved an electromagnetic shielding effectiveness of up to 107 dB over an ultrabroad frequency range of 1–18 GHz. Furthermore, by adjusting the nitrogen doping concentration (3.6–6.3 at%), the shielding performance can be finely tuned (Figure 9c), outperforming conventional metal-based shielding materials. This material holds great potential for applications in electromagnetic protection of flexible electronics, anti-surveillance, radiation shielding, and military stealth technologies [66,67,68].

### 4.3. Flexible Touch Screens

Graphene-skinned glass exhibits high carrier mobility and low sheet resistance while maintaining approximately 82% visible light transmittance, demonstrating excellent electrical performance. The directly grown graphene forms a strong interfacial bond with the substrate, ensuring stable electrical responses under repeated bending and touch operations, highlighting its great potential as a transparent conductive electrode for flexible touch screens.

**Figure 10:**
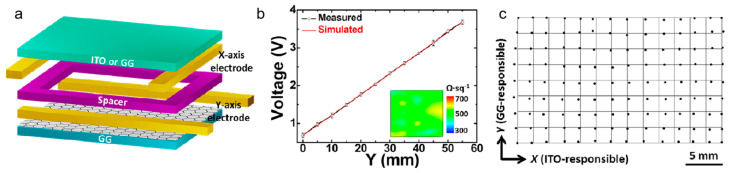
(**a**) Schematic illustration of a graphene-skinned glass touch panel structure. (**b**) Linearity test and sheet resistance mapping of the graphene-skinned glass touch panel. (**c**) Linearity test results of the fabricated touch panel device. Reproduced with permission from Ref. [69]. Copyright 2016, American Chemical Society.

Sun et al. [69] employed methane (CH_4_) as the carbon source in an Ar/H_2_ mixed atmosphere and used a copper-vapor-assisted CVD method at 1050–1100 °C to grow large-area, continuous, and uniform graphene films on quartz glass. By precisely controlling the methane flow rate, growth time, and temperature, a graphene coverage layer with high transparency (82%) and low sheet resistance (370–510 Ω·sq^−1^) was achieved on a 4.5-inch glass substrate. As shown in Figure 10a, the schematic diagram illustrates the touch panel structure. Figure 10b shows the mapping results, confirming the excellent uniformity of the film. Figure 10c presents the linearity test of the touch panel device, demonstrating its high-performance characteristics. This material exhibits extremely low linearity error (1.3%) in touch applications and can achieve rapid defrosting of the glass surface within 180 s under 12 V. Based on this design, researchers have developed an integrated device combining touch sensing and electrothermal defrosting (SGG-based touch panel and defroster), effectively overcoming the limitations of conventional ITO materials, including high cost, brittleness, and difficulty in functional integration. This provides a new materials solution for new transparent conductive glass in smart interactive windows, automotive displays, and aerospace applications.

### 4.4. Optical Fiber Communication

Graphene possesses high carrier mobility, excellent current-carrying capacity, and superior thermal conductivity, making it ideal for high-frequency and high-power optoelectronic applications. Despite being only an atomic layer thick, a single graphene layer can absorb ~2.3% of incident light, and the absorption of multilayer graphene increases approximately linearly with the number of layers. This allows flexible tuning of light absorption by controlling the number of graphene layers [7,70]. Moreover, graphene’s electrically tunable optical properties enable graphene-skinned materials to achieve modulation efficiencies far exceeding those of conventional optoelectronic materials.

**Figure 11:**
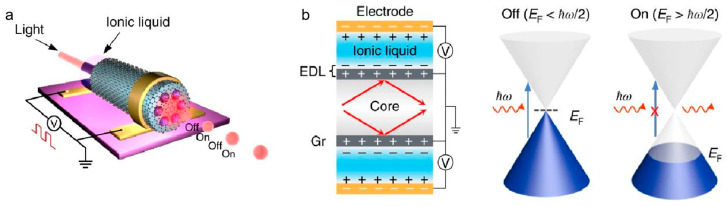
(**a**) Schematic of a graphene-skinned photonic crystal fiber (Gr–PCF)-based electro-optic modulator. (**b**) Working principle of the Gr–PCF electro-optic modulator. Reproduced with permission from Ref. [71]. Copyright 2019, Springer Nature.

Chen et al. [71] employed methane as a carbon source and grew high-quality graphene films in situ on the inner and outer surfaces of photonic crystal fibers (PCFs) via low-pressure chemical vapor deposition under an Ar/H_2_ atmosphere at 1100 °C. By precisely controlling the gas pressure (500–1000 Pa), gas flow rates, and growth time, uniform and continuous graphene coverage was achieved along half-meter-long fiber inner walls, forming a graphene–photonic crystal fiber (Gr–PCF) composite structure. This structure significantly enhances light–graphene interaction while maintaining the waveguiding characteristics of the fiber, achieving an optical attenuation as high as 8.3 dB·cm^−1^. Based on this material, an ion-liquid-gated electro-optic modulator was successfully developed (Figure 11a). The working principle is illustrated in Figure 11b. By electrically tuning the Fermi level of graphene via the ionic liquid, interband absorption can be switched “on” or “off” depending on whether the photon energy is below or above half the Fermi energy. The device exhibits broadband wavelength response (1150–1600 nm) and high modulation depth (~20 dB·cm^−1^ at 1550 nm) under low voltage (~2 V), demonstrating great potential for all-fiber communication systems and integrated photonic devices.

### 4.5. Physiological Electrodes

**Figure 12:**
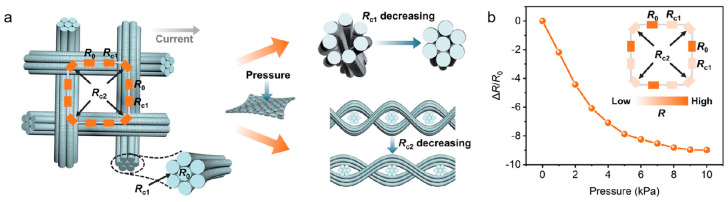
(**a**) Schematic illustration of the GGFF flexible pressure sensors. (**b**) Relative resistance variation under different applied pressures, indicating contact resistance between the warp and weft yarns. Reproduced with permission from Ref. [72]. Copyright 2024, Springer Nature.

Graphene-skinned glass fiber fabrics (GGFF) exhibit excellent electrical conductivity due to their unique woven structure composed of warp and weft yarns, with each yarn containing thousands of individual fibers. This hierarchical conductive architecture enables GGFFs to display ultra-sensitive resistance responses under applied pressure. Consequently, GGFFs are highly suitable as lightweight, flexible pressure sensors with high sensitivity and portability, capable of monitoring human motion and physiological signals such as pulse and acoustic signals. After in situ graphene growth on the glass fiber fabric substrate, a three-dimensional conductive network is formed, where graphene-coated fibers act as conductive pathways. Based on the innovative work by Wang et al. [72], a rapid CVD strategy using dichloromethane (CH_2_Cl_2_) as a precursor was developed. The core breakthrough lies in the dual synergistic mechanism of CH_2_Cl_2_: the low C–Cl bond energy significantly lowers the decomposition barrier, efficiently generating active carbon species in the gas phase; simultaneously, the highly electronegative Cl radicals produced during decomposition enhance carbon adsorption stability on the SiO_2_ substrate via the Cl–CH_2_ co-adsorption effect (adsorption energy reduced by 2.0 eV) and enable zero-barrier H desorption at graphene edges, synergistically accelerating nucleation, domain growth, and coalescence. This approach increases the graphene growth rate by three orders of magnitude (full coverage achieved in 30 s) and improves carbon utilization by 960 times. As shown in Figure 12a, the resulting graphene-skinned glass fiber fabric features a hierarchical conductive network, including intrinsic fiber resistance, inter-fiber contact resistance, and inter-yarn contact resistance. Mechanical deformation modulates both the number of contact points and the contact pressure, producing highly sensitive resistance responses. The GGFF sensor successfully monitors real-time human motion (finger bending response <100 ms) and captures physiological signals with high fidelity, as illustrated in Figure 12b, which shows significant variation in warp–weft contact resistance under different pressures. Thanks to its lightweight (2.5 g·cm^−3^) and high sensitivity (detection limit <10 Pa), this material platform provides a reliable foundation for scalable fabrication of wearable health-monitoring devices and human–machine interaction systems.

### 4.6. Electrothermal Deicing

Graphene-skinned materials combine the flexibility of the substrate morphology with the excellent electrothermal conversion properties of graphene, exhibiting rapid thermal response and highly uniform heat distribution. Therefore, these materials show significant potential for active thermal management applications in extreme environments.

**Figure 13:**
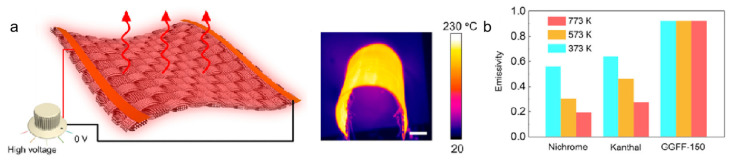
(**a**) Schematic of the GGFF heater. (**b**) Emissivity comparison of commercial metal resistance wires and GGFF-150 at 373 K, 573 K, and 773 K. Reproduced with permission from Ref. [63]. Copyright 2022, American Chemical Society.

As shown in Figure 13a, the GGFF demonstrates outstanding electroheating performance, achieving a heating rate of ~190 °C·s^−1^ at a power density of ~9.3 W·cm^−2^, with temperature non-uniformity below 3% upon reaching saturation [63]. Moreover, graphene-skinned glass fibers exhibit excellent infrared emissivity, with graybody-like characteristics and an emissivity as high as ~0.92 [73,74]. Compared with conventional electroheating materials such as Fe–Cr and Ni–Cr alloys, GGFFs show ultrahigh electrothermal conversion efficiency, with experimental values reaching ~94%. Hao Yuan et al. [63] employed CVD at 1100 °C to directly grow graphene on glass fiber surfaces, successfully fabricating lightweight, flexible dual-emitter infrared electrothermal materials (GGFFs). By precisely controlling the CH_4_/H_2_ gas ratio, the graphene thickness (10–40 nm) and sheet resistance (1–1000 Ω·sq^−1^) were tailored, enabling batch production of large-area fabrics. The resulting materials achieved high infrared emissivity (wavelength-independent, 0.92), thermal radiation efficiency of 79.4%, ultrafast electrothermal response (heating rate of 190.7 °C·s^−1^ at 9.3 W·cm^−2^, saturation temperature 562.0 °C), uniform temperature distribution (±6.1 °C), and excellent mechanical stability (electrical resistance nearly unchanged after 100 cycles of 180° bending). Compared with conventional Ni–Cr heating wires, GGFFs reduce energy consumption by 33.3% and shorten heating times by 8 min in coating-drying applications. Additionally, the synergistic dual-emission effect between the Si–O vibrational modes of the glass fibers and graphene lattice vibrations further enhances radiative performance. As shown in Figure 13b, the spectral radiance of GGFFs is significantly higher than that of commercial metal resistance wires, and its high emissivity remains stable across different temperatures. These results demonstrate that graphene-skinned glass fiber materials hold great promise for active thermal management in extreme environments.

The reviewed applications highlight the potential of graphene-skinned materials to overcome limitations of conventional graphene composites. However, systematic studies quantifying the contribution of interfacial bonding, layer uniformity, and intrinsic graphene properties to device performance are scarce. Future work should focus on establishing performance benchmarks, optimizing growth strategies for diverse substrates, and exploring integration into practical devices at scale.

When compared with other graphene-based composites, graphene-skinned materials exhibit several distinct advantages that stem from their structural characteristics rather than merely compositional differences. Traditional graphene composites—such as those incorporating three-dimensional porous frameworks, reduced graphene oxide networks, or graphene–polymer hybrids—often exhibit performance governed by complex synergistic effects between graphene and the bulk matrix. These intertwined effects make it challenging to decouple and quantitatively evaluate the intrinsic contribution of graphene. In contrast, graphene-skinned systems feature continuous, large-area, and highly crystalline graphene films conformally covering the substrate surface. This configuration allows the intrinsic superiorities of graphene—such as high carrier mobility, exceptional in-plane conductivity, and outstanding mechanical strength—to be fully expressed at the interface, where they most effectively enhance surface-dominated functionalities. Therefore, although direct numerical comparison across different graphene architectures is inherently nontrivial due to their varied morphologies and mechanisms, graphene-skinned materials represent a distinct and complementary strategy. By preserving graphene’s structural integrity and ensuring efficient interfacial coupling, these systems unlock the full potential of graphene’s intrinsic properties for targeted, high-performance surface applications.

## 5. Outlook

In summary, graphene-skinned materials represent a transformative concept bridging two-dimensional materials and bulk substrates. By integrating graphene’s intrinsic properties through direct, transfer-free growth, this emerging platform opens new avenues for high-performance, multifunctional, and scalable material systems across electronics, energy, and structural applications. As an emerging graphene composite system, graphene-skinned materials exhibit remarkable advantages in thermal management, EMI shielding, flexible touchscreens, physiological sensing, and electrothermal deicing applications. Their key strength lies in the ability to directly grow continuous and uniform graphene films on conventional substrates, effectively addressing the challenges of self-support in ultrathin graphene and defects induced by metal substrate transfer. Meanwhile, these materials leverage the structural stability of the substrates, achieving synergistic enhancement of mechanical, electrical, and thermal properties. This endows graphene-skinned materials with significant performance benefits in flexible electronics, aerospace, wind energy, industrial heating, and lithium-ion batteries, with particularly notable breakthroughs in EMI shielding and thermal management.

Currently, CVD is considered the most effective route for producing high-quality graphene-skinned materials. It enables large-area, structurally controllable, and performance-uniform fabrication. By precisely tuning the atmosphere, temperature, pressure, and carbon source, we can obtain graphene films with high coverage, excellent crystallinity, and uniform numbers of layers, ensuring stable performance in electrothermal conversion, optical transparency, and EMI shielding applications. Additionally, strategies such as multi-species co-adsorption, surface catalysis, or plasma-assisted growth provide effective approaches to further increase growth rates, reduce fabrication temperatures, and enhance interfacial adhesion. Alternative fabrication methods—including solution coating and low-temperature in situ reduction—are also under active development for flexible substrate coverage, cost-effective processing, or large-scale continuous production. While each method has its advantages, common challenges remain regarding material uniformity, interface adhesion, and long-term stability.

Despite the significant progress and demonstrated advantages, graphene-skinned materials face several critical challenges before industrial-scale deployment. First, scaling up the fabrication process while maintaining high film uniformity and crystallinity, particularly over complex geometries, remains difficult. Second, the intrinsic stability of the graphene film and its interface with the substrate under thermal stress and environmental factors must be addressed to ensure long-term reliability. Third, standardized performance evaluation methods and quality control protocols are currently lacking, hindering widespread application and integration into industry.

Future research should focus on optimizing CVD processes to improve large-area consistency and batch production capacity. Low-cost and scalable fabrication methods need to be explored to extend applications to complex substrates and flexible devices. It is also important to develop ultrathin EMI shielding layers that combine high shielding efficiency with optical transparency for flexible electronics. Designing lightweight electrothermal deicing structures for aerospace applications can enable active anti-icing and de-icing. Integrating thermal management with electromagnetic compatibility in high-power electronic devices will further enhance performance. With continued advancement in fabrication techniques and application research, graphene-skinned materials are expected to achieve widespread utilization in electronics, energy, and aerospace, representing a key breakthrough for graphene industrialization.

## Data Availability

The original contributions presented in this study are included in the article. Further inquiries can be directed to the corresponding authors.
